# Supplementary material for: Peanut Shell Extract Improves Mitochondrial Function in db/db Mice via Suppression of Oxidative Stress and Inflammation
Source: Nutrients. 2024 Jun 21;16(13):1977. doi: 10.3390/nu16131977 (PMC11243022; doi:10.3390/nu16131977)
Supplement: Supplementary file 1 [file nutrients-16-01977-s001.zip › nutrients-3029301-supplementary.pdf]

Table S1. List of primers for mRNA gene expression

Genes	Forward	Reverse
DRP1	5’-ACA ACA GGA GAA GAA AAT GGA GTT G-3’	5’-AGA TGG ATT GGC TCA GGG CT-3’
FIS1	5′-CTG CGG TGC AGG ATG AAA GAC-3′	5′-GGC GTA TTC AAA CTG CGT GCT-3′
MFN1	5′-AGC TCG CTG TCA TTG GGG AG-3′	5′-TCC CTC CAC ACT CAG GAA GC-3′
MFN2	5’-TCC TGA ACA ACC GCT GGG AT-3’	5’-GAT CCA CCA CGC CTA GCT CA-3’
OPA1	5’-CAG CTG GCA GAA GAT CTC AAG -3’	5’-CAT GAG CAG GAT TTT GAC ACC -3’
PGC-1α	5′-CAG GAG CTG GAT GGC TTG GG-3′	5′-GGG CAA AGA GGC TGG TCC T-3′
TFAM	5′-GCT TCC AGG GGG CTA AGG ATG-3′	5′-TCG CCC AAC TTC AGC CAT TT-3′
PINK1	5′-TCG GCC TGT CAG GAG ATC CA-3′	5′-CAT TGC AGC CCT TGC CGA TG-3′
NRF2	5’-CTC TCT GGA GAC GGC CAT GAC T-3’	5’-CTG GGC TGG GGA CAG TGG TAG T-3’
TNFα	5′-GAA CTC CAG GCG GTG TCT GT-3′	5′-CTG AGT GTG AGG GTC TGG GC-3′
IBA1	5’-GAG CTA TGA GCC AGA GCA AGG ATT T-3’	5’-ACT CCA TGT ACT TCG TCT TGA AGG-3’
β-actin	5′-ACA ACC TTC TTG CAG CTC CTC C-3′	5′-TGA CCC ATA CCC ACC ATC ACA-3′

Abbreviations: DRP1, Dynamin-related protein 1; FIS1, fission 1 protein; MFN1, mitofusin 1; MFN2, mitofusin 2; OPA1, mitochondrial dynamin like GTPase; PGC-1α, peroxisome proliferator-activated receptor gamma coactivator 1 alpha; TFAM, mitochondrial transcription factor A; PINK1, (PTEN)-induced putative kinase 1; NRF2, nuclear factor erythroid 2–related factor 2; TNFα, Tumor necrosis factor alpha; IBA1, Ionized calcium-binding adaptor molecule 1.

Table S2. Summary of antibody dilutions and conditions used in western blot analysis

Primary Antibody	Species	Dilution/Cat. no	Vendor	Secondary antibody	Dilution/ Cat no.	Vendor
DRP1	Rb mAbs	1:1000 / ab184247	Abcam, Cambridge, MA	Gt anti-Rb HRP	1:2000 /7074s	Cell Signaling Technology, Inc., Danvers, MA
FIS1	Rb pAbs	1:1000 / 10956-1-AP	Protein Tech Group, Inc., Chicago, IL	Gt anti-Rb HRP	1:2000 /7074s	Cell Signaling Technology, Inc., Danvers, MA
MFN1	Rb pAbs	1:1000 / NBP1-51841	Novus Biological, Littleton, CO	Gt anti-Rb HRP	1:2000 /7074s	Cell Signaling Technology, Inc., Danvers, MA
MFN2	Rb pAbs	1:1000 / 9482S	Cell Signaling Technology, Inc., Danvers, MA	Gt anti-Rb HRP	1:2000 /7074s	Cell Signaling Technology, Inc., Danvers, MA
OPA1	Rb pAbs	1:1000 / NB110-55290SS	Novus Biological, Littleton, CO	Gt anti-Rb HRP	1:2000 /7074s	Cell Signaling Technology, Inc., Danvers, MA
PGC-1α	Rb pAbs	1:1000 / NBP1-04676	Novus Biological, Littleton, CO	Gt anti-Rb HRP	1:2000 /7074s	Cell Signaling Technology, Inc., Danvers, MA
TFAM	Rb pAbs	1:1000 / ab131607	Abcam, Cambridge, MA	Gt anti-Rb HRP	1:2000 /7074s	Cell Signaling Technology, Inc., Danvers, MA
PINK1	Rb pAbs	1:500 / BC100-494	Novus Biological, Littleton, CO	Gt anti-Rb HRP	1:2000 /7074s	Cell Signaling Technology, Inc., Danvers, MA
NRF2	Rb pAbs	1:500 / NBP1-32822	Novus Biological, Littleton, CO	Gt anti-Rb HRP	1:2000 /7074s	Cell Signaling Technology, Inc., Danvers, MA
TNFα	Rb pAbs	1:1000 / ab6671	Abcam, Cambridge, MA	Gt anti-Rb HRP	1:2000 /7074s	Cell Signaling Technology, Inc., Danvers, MA
IBA1	Rb mAbs	1:1000 / Ra17198S	Cell Signaling Technology, Inc., Danvers, MA	Gt anti-Rb HRP	1:2000 /7074s	Cell Signaling Technology, Inc., Danvers, MA
B-actin	Mu mAbs	1:2000 /A2228	Millipore Sigma (Burlington, MA, USA)	Ho anti-Mu HRP	1:2000 /7076s	Cell Signaling Technology, Inc., Danvers, MA

Abbreviations: DRP1, Dynamin-related protein 1; FIS1, fission 1 protein; MFN1, mitofusin 1; MFN2, mitofusin 2; OPA1, mitochondrial dynamin like GTPase; PGC-1α, peroxisome proliferator-activated receptor gamma coactivator 1 alpha; TFAM, mitochondrial transcription factor A; PINK1, (PTEN)-induced putative kinase 1; NRF2, nuclear factor erythroid 2–related factor 2; TNFα, Tumor necrosis factor alpha; IBA1, Ionized calcium-binding adaptor molecule 1, Rb-Rabbit, Mu- Mouse, Gt- Goat, Ho-Horse, mAbs-Monoclonal antibody, pAbs-Polyclonal antibody.
